# How can social robot use cases in healthcare be pushed - with an interoperable programming interface

**DOI:** 10.1186/s12911-023-02210-7

**Published:** 2023-07-11

**Authors:** Robin Glauser, Jürgen Holm, Matthias Bender, Thomas Bürkle

**Affiliations:** Institute for Medical Informatics BFH, Berne University of Applied Sciences, Höheweg 80, CH 2502 Biel, Switzerland

**Keywords:** Interface, Robotics, Healthcare, Python, Middleware, Websocket, ROS, Pepper, Cruzr, Teaching, Java, Students, Softbanks, Ubtech, Android

## Abstract

**Introduction:**

Research into current robot middleware has revealed that most of them are either too complicated or outdated. These facts have motivated the development of a new middleware to meet the requirements of usability by non-experts. The proposed middleware is based on Android and is intended to be placed over existing robot SDKs and middleware. It runs on the android tablet of the Cruzr robot. Various toolings have been developed, such as a web component to control the robot via a webinterface, which facilitates its use.

**Methods:**

The middleware was developed using Android Java and runs on the Cruzr tablet as an app. It features a WebSocket server that interfaces with the robot and allows control via Python or other WebSocket-compatible languages. The speech interface utilizes Google Cloud Voice text-to-speech and speech-to-text services. The interface was implemented in Python, allowing for easy integration with existing robotics development workflows, and a web interface was developed for direct control of the robot via the web.

**Results:**

The new robot middleware was created and deployed on a Cruzr robot, relying on the WebSocket API and featuring a Python implementation. It supports various robot functions, such as text-to-speech, speech-to-text, navigation, displaying content and scanning bar codes. The system’s architecture allows for porting the interface to other robots and platforms, showcasing its adaptability. It has been demonstrated that the middleware can be run on a Pepper robot, although not all functions have been implemented yet. The middleware was utilized to implement healthcare use cases and received good feedback.

**Conclusion:**

Cloud and local speech services were discussed in regard to the middleware’s needs, to run without having to change any code on other robots. An outlook on how the programming interface can further be simplified by using natural text to code generators has been/is given. For other researchers using the aforementioned platforms (Cruzr, Pepper), the new middleware can be utilized for testing human-robot interaction. It can be used in a teaching setting, as well as be adapted to other robots using the same interface and philosophy regarding simple methods.

## Introduction

### Topic and goal of the research

Robots are making their way into everyday life and healthcare [1]. In medicine, robots are used for e.g. logistical use cases [2] and robot-assisted surgery [3].

In addition, social robots are discussed [4] regarding their use for patient care. Robots like Pepper or Paro are being used in elderly care homes [5] and for social robots use cases [6–8] e.g. for medication reminders, entertainment purposes or hygiene tasks. During the Covid-19 pandemic, robots were even used for scanning patients’ temperatures in hospitals and tele-medicine services for infected people [9–11].

Rapid adaption to new use cases is necessary for the utilization of robots in medical environments such as the control of patient flows during a pandemic. Typical robotic activities such as moving around, moving robotic arms, language understanding and language output need to be abstracted and simplified. Thus, the implementation of new use cases should be easy and rapid, so that it can be performed by staff in the respective medical environment. These could be the IT workers of the institution or technically minded healthcare staff who define and adapt concrete use cases for their working environment.

Current robot programming solutions such as robot operating system ROS [12] seem too complicated and require considerable time and knowledge to master.

Therefore, the development of a robot middleware has been started to facilitate the implementation of social robotics use cases in a fast and simple manner, while being relatively independent of the existing and future robot hardware.

The future options for using robots in medicine will depend upon the ease of adaptation to the respective medical use case. The middleware solution should help to avoid that an IT professional who has experience with robots is required each time a medical use case is being changed.

### Why is a new methodology necessary?

Several robot middleware solutions have been devised to simplify programming of robots [12], e.g. “Robot Operating System” ROS [13]. ROS is a software invented by the Stanford Artificial Intelligence Laboratory with the goal to support robot manufacturers to create reusable modules and algorithms that can be used on different hardware. Other such technicaloriented computing middleware include programming robots are OpenRDK [14], Yarp [15], and Orca [16].

While ROS has been adapted by many robot manufacturers, e.g. Cruzr, the other mentioned middleware solutions are either outdated or not widely adopted. However, most middleware solutions are still technically oriented and focus on solving logistical issues, which makes them difficult to use for persons with limited programming experience. E.g. setting up robot navigation within ROS requires first the setup of a navigation stack which combines sensor streams with movements. Thus, advanced IT and robotics skills/expertise, as well as considerable programming skills are necessary to implement a medical use case such as reception and guiding of a patient to a department.

On the other hand, there are some graphical programming interfaces such as Choregraphe Suite. Such solutions are often supplied by a single vendor and proprietary for the robots of this manufacturer. They have been successfully employed for teaching use cases, but they are limited in their abilities. There is also a risk that they are outdated or insufficiently supported when new hardware becomes available.

Therefore, it is desirable to provide a neutral middleware solution which abstracts commands specific for one type of hardware to generic actions such as moveTo(10,23) or say(“Hello, I am a robot”) and can be used on different hardware.

### The working environment: robots and software

This paper refers to two types of robots. The Cruzr^1^ robot is based on two operating systems working together. The head uses Android 5.1.1^2^ to provide a touchscreen interface. The base uses ROS and Ubuntu 18.04 to control the arm motors and to provide the navigation functionality. However, the access to this second system was not provided by the manufacturer at the time of developing the middleware.

The other robot is a Pepper 1.8a^3^ running Naoqi 2.5.7.1, a Gentoo based operating system, in the head and Android 5.1 on the attached tablet. Cruzr came with a software SDK which is only usable with the outdated version Android 5.1.1. For Pepper, the Choregraphe Suite^4^ was available but was outdated and out of support as well.

The middleware described here was initially implemented for the Cruzr robot but has been extended to run on the Pepper robot as well.

## Methods

**Fig. 1:**
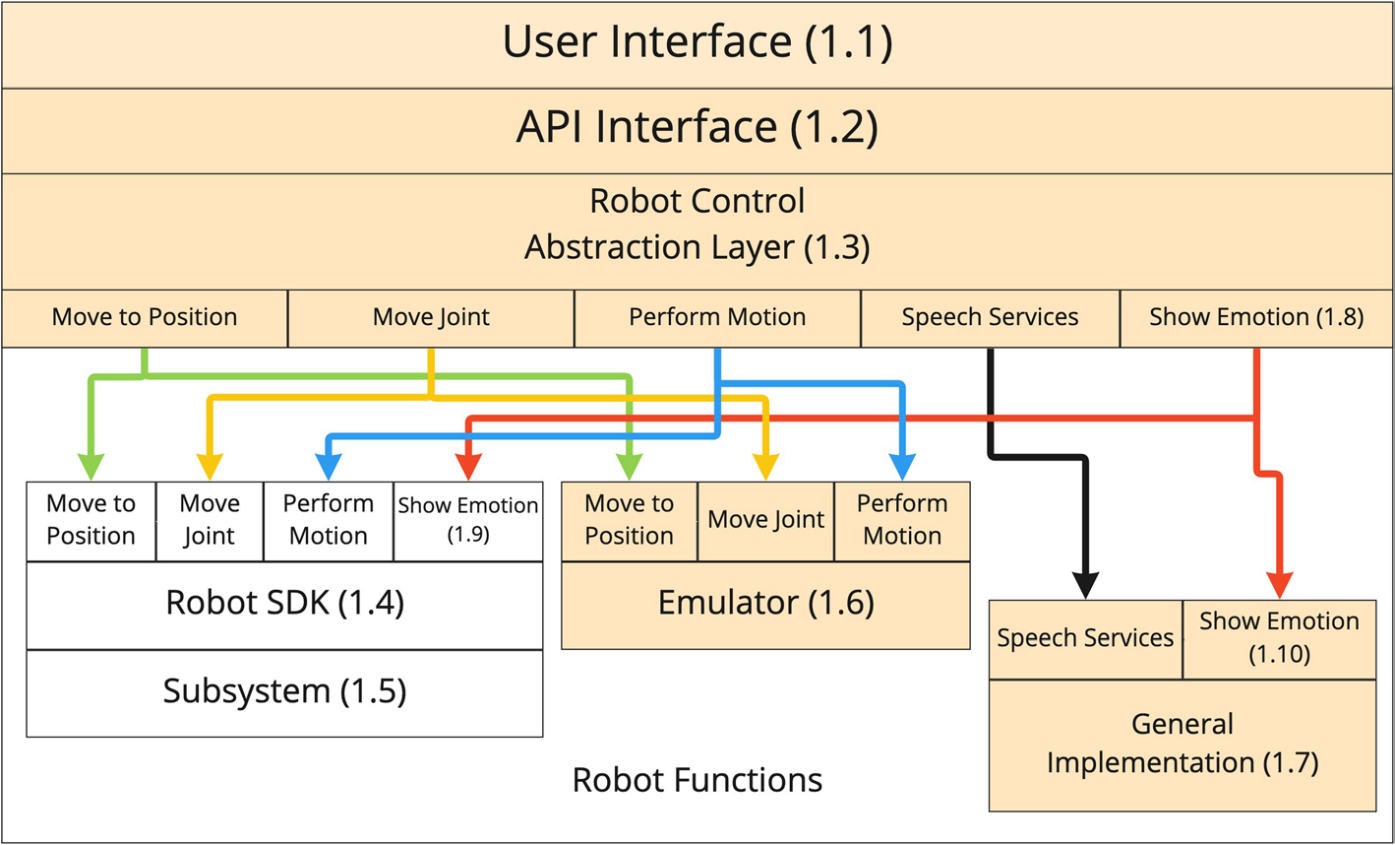
The Abstract Architecture Diagram shows the different layers enabling the new middleware. At the top layer is the user interface (1.1) with which the robot can be programmed. It accesses the Robot Control Abstraction Layer (1.3) via the API Interface (1.2). The Robot Control Abstraction Layer (1.3) enables the use of the different robot SDK’s (1.4) so one use case can be run on multiple robots. The SDK’s can then use their subsystems (1.5) to carry out the task. Depending on which robot the middleware is run, it dispatches the functions to the relevant SDK (1.4) or general implementations (1.7). This architecture enables an emulated robot (1.6) on a normal tablet to test code without using a robot, by implementing dummy functions on an emulator robot SDK. It’s possible to reuse general implementations (1.7) across multiple robots, for example for showing emotions on a tablet (1.10), (1.8). If a robot implements general features like showing emotions (1.9), the robot’s function can be used instead of the general implementation (1.10)

The middleware is based on an application programmer’s interface (Fig. 1.1), an abstraction layer which translates generic robot actions (Fig. 1.3) and one or several robot interfaces (Fig. 1.4) to transmit the respective actions to each type of robot (Fig. 1). Starting with the Cruzr robot interface which uses Android version 5.1.1 the different robot actions were studied and implemented in the robot control abstraction layer using the Cruzr SDK.

The different robot actions were then made accessible over a WebSocket interface. For the application programmer’s interface, it was decided to use Python, as it is a widely used language, as well as often used in robotics development [17]. In the end, the new solution should be compared to the existing solutions.

The middleware was implemented as an Android Java app, which runs on the Cruzr tablet. The app provides a WebSocket server with an interface with which the robot can be controlled.

The speech interface was implemented using the Google Cloud Voice text-to-speech and speech-to-text services.^5^. A German vosk language model (vosk-model-small-de-0.15) was utilized for the background “hot word listener”.^6^

A Python implementation was created for this interface, as Python is a language which is easy to pick up. Other languages, which have WebSocket support, can also be used.

Furthermore, a web interface was created to try out the interface, enabling direct control of the robot. This kind of interface can also be run on other robots that include an Android device in their architecture.

The interface can be reimplemented for other platforms, enabling the use of the tooling created for the interface.

The app was created to enable the WebSocket interface on the robot, which included a basic awareness module for demonstrations. A simple web application, utilizing jQuery, was implemented to test the WebSocket interface.

Documentation for the interface was written with Mkdocs with the Material theme^7^ in Markdown, with examples included to facilitate use of the interface, allowing user to focus on implementation of healthcare use cases.

Implementations of the basic functions were created with the WebSocket interface, using the Python language.

## Results

**Fig. 2:**
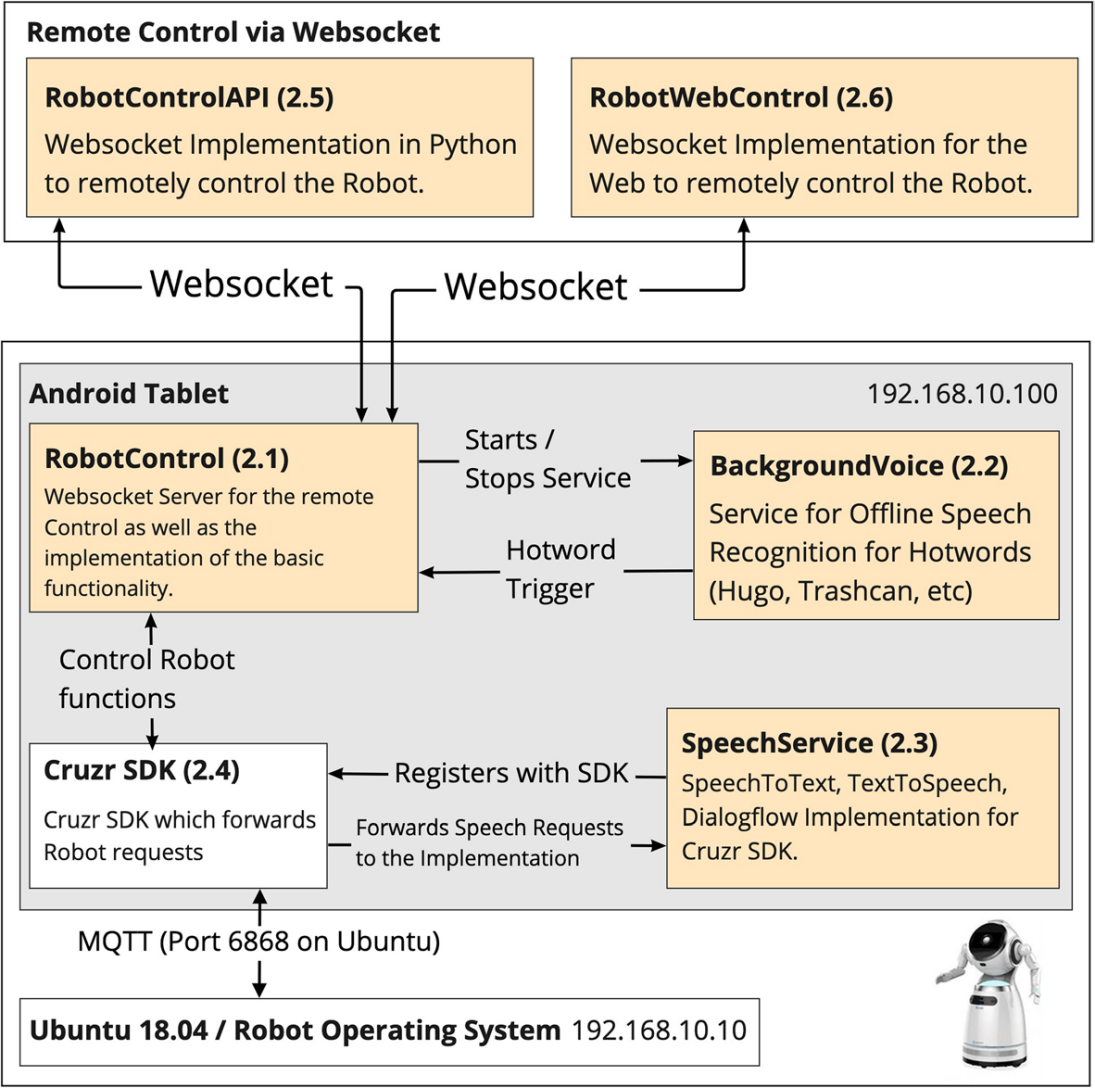
The architecture overview shows the final implementation of the abstract architecture for the Cruzr robot. A WebSocket interface (2.1) called RobotControl, a service to recognize hotwords called BackgroundVoice (2.2), a service for text-to-speech and speech-to-text via Google cloud services with a dialogflow implementation called SpeechService (2.3), the Cruzr SDK for Android (2.4) which can be replaced by another robot SDK, the Python implemention for the WebSocket interface called RobotControlAPI (2.5) and a webinterface to test out robot functions called RobotWebControl (2.6)

The new middleware is depicted in (Fig. 2). It is based on multiple components:

The main component and heart of the middleware is the RobotControl component (Fig. 2.1). It contains the WebSocket server, the implementation of the functions, and provides an interface to interact with and to control the robot it is run on.

The BackgroundVoice (Fig. 2.2) component is an additional component that provides a way to listen for keywords and trigger actions when the robot hears a keyword.

The SpeechService (Fig. 2.3) component is needed for the Cruzr robot, implements the language services for text-to-speech and speech-to-text for the Cruzr robot.

The Cruzr SDK (Fig. 2.4) is used to control the cruzr robot. It was provided by Ubtech, the manufacturer of the Cruzr robot.

The RobotControlAPI (Fig. 2.5) is the WebSocket client implementation in Python to remotely control the robot.

The RobotWebControl (Fig. 2.6) is a web interface to access the WebSocket server and remotely control the robot.

In the next sections, an examination of the middleware components is undertaken in detail.

### RobotControl (2.1)

The interface was implemented to run as an app on Android and provided an API for other programming languages to connect to over the network over a WebSocket interface. This makes it trivial to install on the users’ laptops, as they only need to install Python and the RobotControlAPI library (Fig. 2).

As the underlying technology, HTTP was used in the first prototype and then extended with a WebSocket^8^ interface, which supports real time events and enables the usage of waiting for an action to be done, without having to rely on HTTP long polling.

The interface exposes a web API and runs the code from the Cruzr SDK (Fig. 2.4) to make the robot speak, move, show emotions, listen to the person in front of him or display content on the robot’s tablet.

**Fig. 3:**
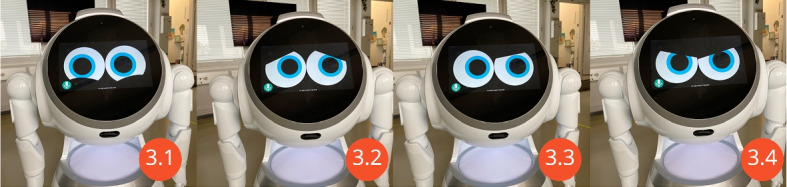
The Cruzr robot can show different emotions using the interface function 8 from Table 1: happy (3.1), sad (3.2), confused (3.3) and angry (3.4). These emotions can be used on other robots with a tablet as a face

The navigation and gesture functions were provided by the Cruzr SDK. The speech functions use Google Cloud Voice text-to-speech and speech-to-text. For the robot face, a custom web page animation was created (Fig. 3) which displays a abstract face which can be animated [18].

The RobotControl app also includes some basic life functions which can be triggered via voice command like going back to the charging station and integration with the SpeechServices to provide basic question answer functions.

The Interface supports the functions listed in (Table 1). Those include making the robot speak, making the robot move to a specific position, listening to what someone in front of the robot is saying, performing gestures, showing emotions and scanning barcodes.

**Table 1:** Interface Functions implemented in the middleware

Nr	Function
1	Making the robot speak
2	Change the robot’s volume
3	Moving the robot around with a joystick like interface
4	Moving the robot around on a map
5	Make the robot go back to the charging station
6	Finding out the current location of the robot
7	Localize the robot, when it is in the wrong location
8	Make the robot show different emotions (Fig. 3)
9	Make the robot perform gestures
10	Move the different servos on the robot
11	Look at the debug log to see errors
12	Use the robot for telepresence
13	Opening a QR / Bar code Scanner on the robot
14	Display web pages on the robot’s display
15	Interact with the web page on the robot’s display
16	Listen for speech and convert it into text

### BackgroundVoice (2.2)

To enable the robot to listen to hot words in the background, a separate component was created that can listen to specified keywords and trigger an action.

This was implemented using the vosk speech-to-text engine based on their android app template^9^.

The vosk-model-small-de-0.15 was included in the app to enable the offline always on speech recognition^10^.

### SpeechService (2.3)

The SpeechService component provides the middleware with the text-to-speech and speech-to-text functions. It uses the Google Cloud services combined with Dialogflow to answer predefined questions. This app was based on a provided template from Ubtech to enable including and modifying the speech services on the Cruzr robot. It was extended to translate system messages from English to German and configured to use a custom Dialogflow model in the Google Cloud for basic answer and question functions.

The robot has a skill to change the language of the robot to English or French via voice commands or a touchscreen interface.

### Cruzr SDK (2.4)

The Cruzr SDK 2.8.0 is a component provided by Ubtech, the manufacturer of the robot. It provides the functions of the robot to navigate, move joints and control the lights of the robots and trigger the speech service which was implemented. The SDK was provided as a Java Archive (.jar), which was included in the RobotControl App as an external library to enable the App to forward actions from the WebSocket.

### RobotControlAPI (2.5)

A Python implementation, which implements functions 1-16 from (Table 1) was created to enable the use of the WebSocket interface from a Python program.

The philosophy behind the interface was based on the book “A Philosophy of Software Design” [19]. The methods were designed to be available through the interface in a simple manner, as described in the book, promising to carry out the action ascribed to them. This means handling simple errors, providing an interface without many parameters and being able to connect to the robot without having to initialize a lot of classes. The interface is based around a class called Robot, which is used to connect to the robot and with which you can send messages to the robot to make it run a command. The Robot class contains methods to generate messages with simple parameters.

With the Python implementation it is possible to program the robot in a generic manner, which can be used for different robots. (Listing 1).

**Figure:**
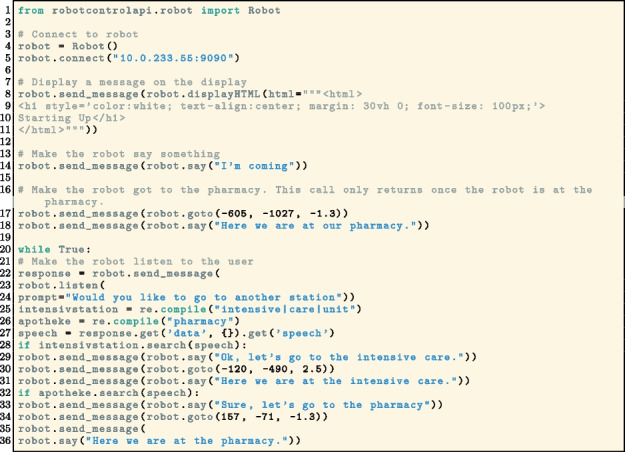
**Listing 1** Example code for RobotControlAPI. It shows how the basic functions speech, navigation, display and speech recognition can be used. The robot will show a starting up message, then say “I’m coming”. Afterwards the robot will move to the pharmacy and say “Here we are at the pharmacy” after arriving. At the pharmacy the robot will ask if the user wants to go to another station. Depending on the answer the robot will move to the mentioned station

This code outlines the various methods of the interface. The robot will first display a message on the tablet, followed by an audible statement of “I’m coming”. Subsequently, the robot will navigate to the pharmacy using coordinates. Upon arrival, the robot will announce “Here we are at our pharmacy”. Finally, the robot will listen to the user and drive to the specified destination, either the pharmacy or the intensive care unit.

**Fig. 4:**
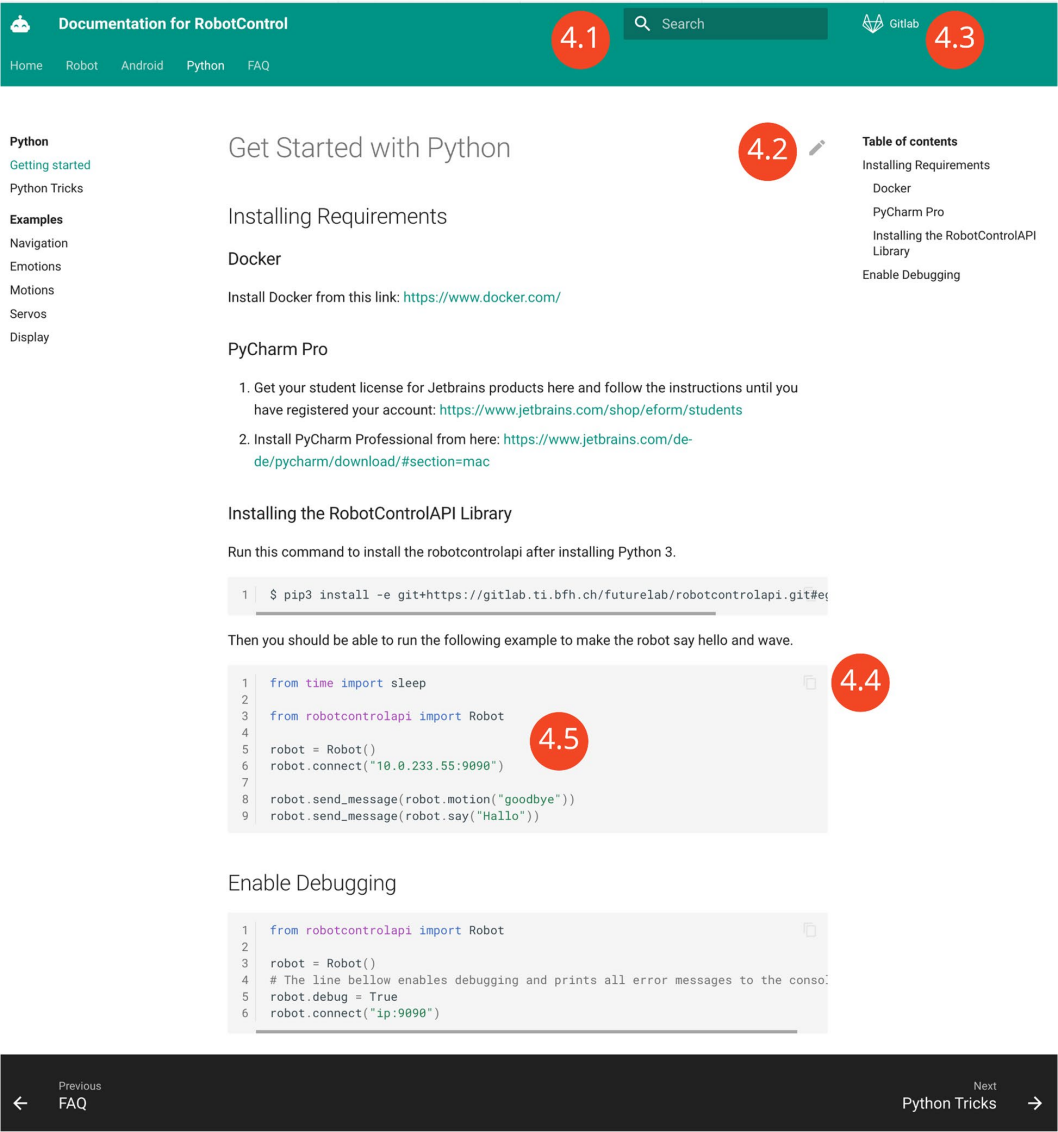
The documentation for the Python Interface (RobotControlAPI) is created with Mkdocs with the Material Theme which is updated with continuous integration. There is a search function to search through the documentation (4.1), a link to edit the page the visitor is on and improve the documentation (4.2), a link to the Gitlab repository (4.3), a button to copy the code in the right formatting (4.4) and a lot of examples to get started with the middleware (4.5). The documentation contains information about the extension of the WebSocket interface, to add new robots or skills to existing robots

The created Python WebSocket interface was documented with Mkdocs Material in markdown (Fig. 4).

This enables the users to learn about the interface individually, makes it easy to add new knowledge by providing direct links to the edit page and updating the mkdocs automatically by using continuous integration^11^. There is a search function to search through the documentation (Fig. 4.1), a link to edit the page the visitor is on and directly improve the documentation (Fig. 4.2), a link to the Gitlab repository (Fig. 4.3), a button to copy the code in the right formatting (Fig. 4.4) and a lot of examples to get started with the middleware (Fig. 4.5).

The interface and the middleware architecture were documented so that new robots can be supported, and new skills can be added.

### RobotWebControl (2.6)

**Fig. 5:**
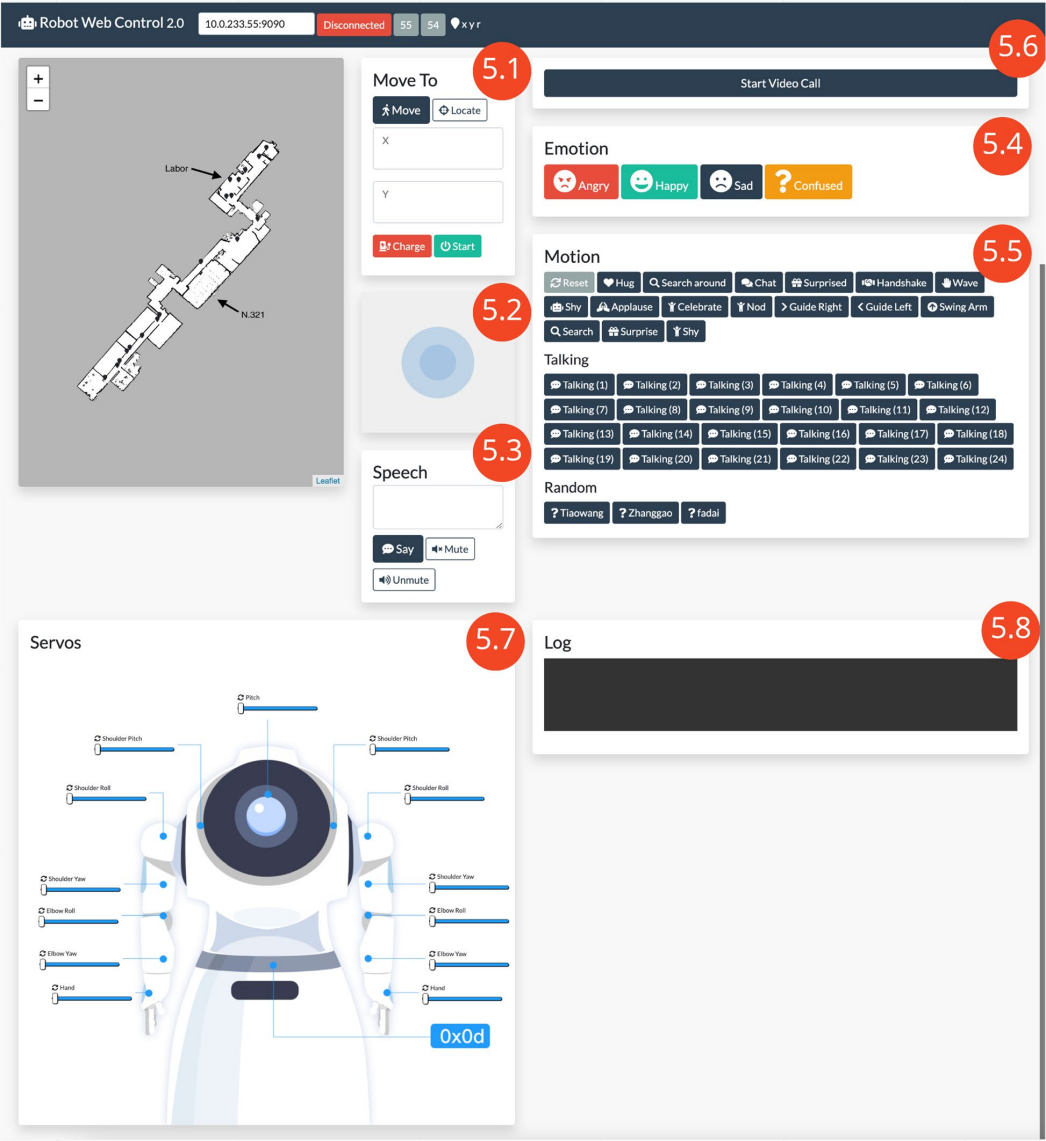
The web interface to control the robot manually (RobotWebControl). The web application enables the usage of functions 1-11 from (Table 1). This includes clicking on a point on the map to get the robot to move to it (5.1), manual joystick control (5.2), a way to make the robot speak out loud (5.3), displaying emotions (5.4), performing different motions (5.5), start a video call (5.6), move the robot’s servos (5.7) and look at the logs for errors (5.8)

As a demonstrator, a web component (Fig. 5) was created to show the functions of the interface and enable an easy demonstration for tours of the BFH laboratory. This is similar to the existing Wizard of Oz Interfaces created for the Pepper Robot [20]. The web application enables the usage of functions 1-12 from (Table 1).

One can click on a point on the map to make the robot move there (Fig. 5.1), move the robot with a joystick (Fig. 5.2), make the robot say something aloud (Fig. 5.3), make the robot perform gestures (Fig. 5.4), make the robot show emotions (Fig. 5.5), starting a video call (Fig. 5.6), moving the robot’s servos (Fig. 5.7) and looking at the logs for errors (Fig. 5.8).

The source code of the application is in a private repository^12^.

### Validation of the results

The first use case for the robot middleware was a new students course for bachelor students in medical informatics. Bern University of Applied Sciences teaches bachelor students in medical informatics since 2011.

In a modernization of the curriculum a new course was established to introduce students to the future perspective of social robot use in medical environments [21]. There, students were given the task to implement a robot guided visitor tour within the medical informatics lab [22]. Students were asked to include additional medical tasks such as recognizing a medication box in this lab tour. The course was given in groups of five students in a one week full time format. Initially, two Cruzr and two Pepper robots were available.

The students utilized the middleware extensively throughout the course without any technical issues. All groups implemented the robot-guided lab tour, as well as additional medical tasks on top of the middleware. On the first day of the course, the students were able to achieve successful movements and interactions with the robot within minutes.

The students feedback was mostly positive concerning the interface and simple design of the API and the tools provided with it.

The following issues were found:

- There were some issues with the servo motors controlling the arms of the cruzr robot. The provided robot API didn’t permit to keep the robot’s arms lifted. The fix was to repeat the command to raise the arm until the next command is sent to the robot.
- The camera located in the robot head couldn’t be used with all functions. There was a malfunction when using the bar code application while trying to take pictures. Therefore, image acquisition was disabled in favor of bar code reading.
- On one occasion, problems with the speech recognition were encountered when using one word sentences containing only numbers. These couldn’t be resolved in the Google Cloud speech engine. There was an unintended learning effect for the students as they realized that robot programming should include different variations e.g. to capture inputs alternatively through the touchscreen.
- The implemented BackgroundVoice component proved impractical due to too much background noise triggering false positives.

## Discussion

### Discussion of results

The strength of the middleware is in its rapid and fast development of practical use cases even for persons with limited programming capabilities. The design of the robot functions do not require any configuration and the commands are straightforward and simple. Complex robot functionality such as moving to a point on a map are reduced to a simple function call.

In comparison with e.g. Choregraphe, complex use cases with multiple feedback loops can be implemented in Python modules, whereas in Choregraphe this leads to incomprehensible scenarios.

Advanced features such as speech recognition, text-to-speech and navigation can be provided to the inexperienced user already at the beginning and permit realistic medical use cases.

The Python programming language can be used easily by users without much programming experience. Current and modern program libraries provided by Python result in a developer friendly programming environment. Code can be exchanged between different groups of programmers.

It is possible as well to implement the interface for other languages than Python, as the provided WebSocket interface is simple to understand and implement.

### Discussion of limitiations

**Fig. 6:**
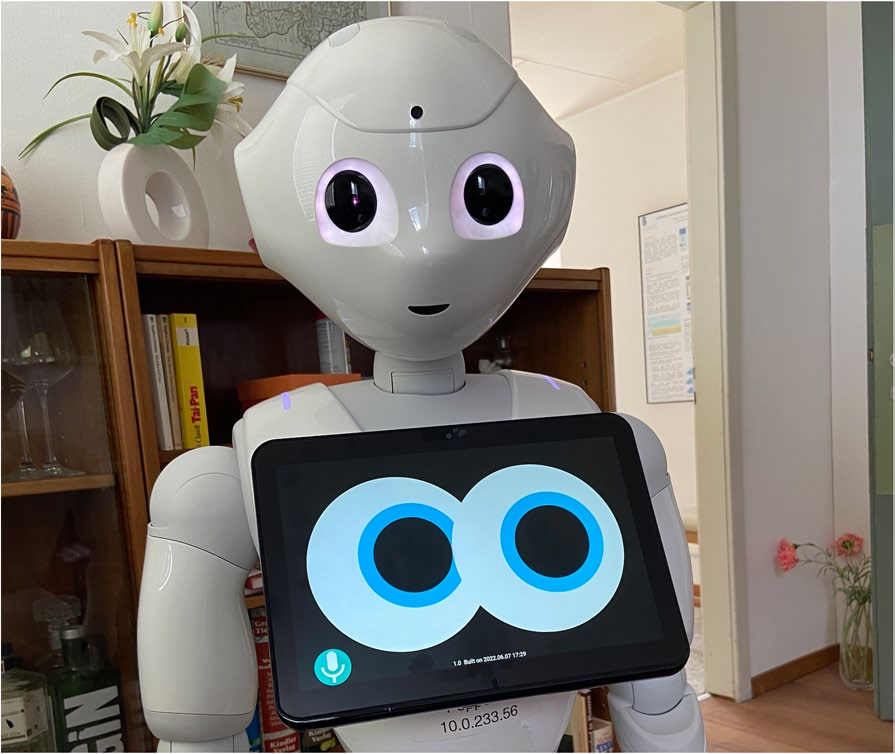
The middleware running on the Pepper robot’s tablet, which runs on Android. This shows that the middleware could be run on multiple robots. To be fully functional, it would be necessary to implement all the skills for the pepper as well, which is technically possible

The middleware was partially ported to the Pepper robot (Fig. 6). This means however that code for moving servos, arms, and specific commands for the Cruzr needs to be abstracted. Instead of moving the arms and servos, the interface needs to provide more abstract methods for listing the objects the robot sees, grabbing one of the objects and placing it in a predefined space.

Adaption to new hardware will require adding functionalities to the middleware e.g. if one wanted to drive a robotic dog a function such sitdown() needs to be implemented. Thus, the function moveTo(x,y) needs to be connected to the robotic dog SDK.

We have used the proposed middleware solution in two student courses over two consecutive years. The students had prior programming experience from their first year of study and completed the course in their second year. It is yet to be determined if the middleware can be used by medical staff with or without prior knowledge.

Furthermore, the middleware may not be suitable for all healthcare use cases due to the limited functions that have been implemented. The design of the middleware functions to be used without requiring any configuration could make it difficult to create more complex use cases.

At the moment robots using this middleware can’t run scenarios without being controlled by an external device, e.g. a laptop. However, this is not a technical limitation and can be addressed in future versions. Furthermore, the presented middleware relies on Android, which may limit its usability with other robots.

### Outlook

Google cloud services were utilized for speech recognition and speech synthesis, which resulted in occasional latency and service outages. Furthermore, a constant internet connection is required.

For medical use cases, it is an open question whether cloud-based voice services can be utilized for sensitive medical information. In the future, it will be necessary to examine whether speech recognition and synthesis should be run on the robot itself, a server on the premises, or as a cloud service. The decision will be contingent upon the uptime and reliability of the services.

In this project, the Python programming language has been successfully utilized with the middleware. It is conceivable to envision a future where natural language commands, such as “Robot, please bring the garbage to the bin,” are sufficient for interaction. To reach such an advanced state, intermediate steps such as instructing the robot by demonstrating the tasks may be necessary [3]. This could eventually eliminate the need for programming languages such as Python. Similarly, code generating environments which are given an oral task description, such as OpenAI Codex^13^ or GitHub Copilot^14^ can be employed in conjunction with robots. This could reduce the barrier for people to use robots in everyday settings, such as hospitals and nursing homes.

### Can other researchers, based on the particular paper, reuse the method?

With this kind of middleware, it is possible to easily adapt to new use cases without having robotics knowledge. The simple design makes it easy as well to create new use cases. The middleware enables human robot interaction tests, with the web interface or with simple scripts using the Python implementation.

At the present time, the source code and documentation have not yet been published. The middleware and other components are in a private repository of the Bern University of Applied Sciences^15^^,^^16^^,^^17^^,^^18^. Those who are interested in using the middleware can contact us.

Our contribution allows other researchers to adapt their robots to the middleware or use the Cruzr robot with the middleware to enable an easier adaption of their use cases.

The course shows that it is possible to build a simplified robot middleware that can be used to build social robotics healthcare use cases and adapt them to new challenges.

## Conclusion

The following three points highlight the important aspects of this research paper:

1. This research paper shows that the new middleware can enable students or non-robotics experts to implement simple to intermediate use cases within a short time span.
2. It demonstrates that the middleware can run on multiple robots and shows ways how the same code can run on different robots by using an abstraction of robot movements.
3. New ideas to make programming a robot easier are discussed, along with the possible concepts that can be realized with current natural language processing (NLP) technology.

## Availability and requirements

 

- **Project name:** RobotControl
- **Project home page:** Not available
- **Operating system(s):** Android 5.1.1
- **Programming language:** Java, Python, Javascript
- **Other requirements:** A cruzr robot for using the middleware
- **License:** Copyright owner
- **Any restrictions to use by non-academics:** The current implementation is based around the laboratory, but can be customized to fit other environments.

## Data Availability

The source code has not been published yet, as the first version is still custom made for the laboratory. Testing of the robot has already been conducted in other environments, with a demo and access to the code available on premise or through video conference.

## Footnotes

1 https://www.ubtrobot.com/de/products/cruzr?ls=en

2 https://www.android.com/intl/de_de/versions/lollipop-5-0/

3 https://developer.softbankrobotics.com/pepper-naoqi-25

4 Choregraphe Suite 2.5.10.7 available for Linux, Windows and Mac https://developer.softbankrobotics.com/pepper-naoqi-25-downloads-linux

5 https://cloud.google.com/text-to-speech?hl=de

6 https://alphacephei.com/vosk/models

7 https://squidfunk.github.io/mkdocs-material/

8 https://developer.mozilla.org/en-US/docs/Web/API/WebSockets_API

9 https://github.com/alphacep/vosk-android-demo

10 https://alphacephei.com/vosk/models

11 https://squidfunk.github.io/mkdocs-material/publishing-your-site/

12 https://gitlab.ti.bfh.ch/futurelab/robotwebcontrol

13 https://openai.com/blog/openai-codex/

14 https://github.com/features/copilot

15 https://gitlab.ti.bfh.ch/futurelab/robotcontrol

16 https://gitlab.ti.bfh.ch/futurelab/speechservice

17 https://gitlab.ti.bfh.ch/futurelab/backgroundvoice

18 https://gitlab.ti.bfh.ch/futurelab/robotcontrolapi
